# Harnessing fluorescent probes for molecular diagnosis and theranostics of atherosclerosis

**DOI:** 10.1080/10717544.2025.2595728

**Published:** 2026-01-09

**Authors:** Xiang Mao, Xia Zhao, Zhigang Ni, Xinwen Xu, Qiang Liu, Peng Qu

**Affiliations:** a Faculty of Medicine, Dalian University of Technology, Dalian, PR China

**Keywords:** Fluorescent probe, atherosclerosis, targeting, imaging, auxiliary diagnosis

## Abstract

Cardiovascular disease remains a leading cause of morbidity and mortality worldwide, posing a serious threat to human health. Atherosclerosis (AS), the pathological basis of most cardiovascular diseases, is characterized by arterial wall thickening caused by chronic inflammation. In recent years, molecular probes have attracted much attention as versatile tools for the diagnosis and treatment of AS, offering capabilities in imaging, drug monitoring, and surgical navigation. The existing probes include fluorescent probes, SERS probes, nuclear medicine probes, self-assembled nanoprobes, UC-FRET probes, photothermal probes, and multimodal probes. Among them, fluorescent probes have emerged as a research focus because of their excellent targeting effect, biocompatibility, and multimodal compatibility. This review summarizes recent advances in the classification and synthesis of fluorescent probes, their targeted applications in AS, and their auxiliary diagnosis and treatment of AS. By highlighting current progress and key challenges, this work aims to provide valuable insights to support further development and facilitate the advancement of fluorescent probe technologies in the context of AS, while promoting the clinical application of fluorescent probes.

## 1. Introduction

Cardiovascular disease (CVD) has high morbidity and mortality worldwide, posing a serious threat to human health. By 2023, it is estimated that more than 300 million people in China have CVD, including about 270 million patients with hypertension (National Center for Cardiovascular Diseases the Writing Committee of the Report on Cardiovascular Health and Diseases in China 2024). The mortality rate associated with CVDs surpasses that associated with tumors and other diseases, accounting for more than 40% of the deaths among residents (Zhu et al. 2024). Atherosclerosis (AS), the pathological basis of most CVDs, is a disease that causes thickening of the arterial wall due to chronic inflammation (Antonino 2012). Lesions typically involve elastic arteries and large, medium, and small muscular arteries (Wang et al. 2023). AS progresses and occludes the arterial lumen, downstream tissues and organs may suffer from ischemia or necrosis, leading to serious diseases such as myocardial infarction and stroke (Puylaert et al. 2022).

A variety of molecular imaging probes have been developed for the diagnosis and treatment of AS, yet each has notable limitations. For example, surface-enhanced Raman scattering (SERS) probes not only require complex synthesis paths but also limit the depth of imaging in vivo (Deriu et al. 2023; Li 2023). Nuclear medicine probes are hindered by radioactive risk, short half-life, and high cost (Hill et al. 2020; Li 2023). Self-assembled nanoprobes suffer from unstable signal outputs due to microenvironment dependence (such as pH fluctuations), and biosafety is doubtful (Zhao et al. 2014; Dong 2019; Kim 2020). Upconversion fluorescence resonance energy transfer (UC-FRET) probes face challenges such as low energy transfer efficiency and the trade-off between material stability and biocompatibility (Marin 2018; Malhotra 2022), the photothermal conversion of the photothermal probe is uneven, and the intraoperative real-time performance is insufficient (Lee 2008; Deng et al. 2019; Guo 2025). Additionally, integrating multimodal probes is difficult, and the lack of targeting specificity can easily lead to false positives (Wang et al. 2022). In this context, fluorescent probes have emerged as a particularly promising avenue for auxiliary diagnosis and therapy.

Fluorescent probes enable strong detection and real-time in vivo dynamic monitoring of early-stage atherosclerotic plaques through highly specific targeted molecular markers such as inflammatory factors and matrix metalloproteinases. When combined with near-infrared (NIR) or two-photon imaging technologies, they can overcome the resolution and depth limitations of traditional imaging methods, allowing precise visualization at the molecular level and resolution of small lesions (Fuchs et al. 2017; Situ 2019). The fluorescent probe also offer excellent multimodal compatibility and can be combined with imaging platforms such as magnetic resonance imaging (MRI) and computed tomography (CT) to improve diagnostic accuracy (Li et al. 2020; Thankarajan 2023). Techniques such as intravascular fluorescence imaging and confocal microscopy enable minimally invasive acquisition of ultrahigh-resolution images of the vascular wall and cell substructure in a minimally invasive way, facilitating the evaluation of plaque stability and rupture risk. In addition to their use in diagnostics, fluorescent probes have the potential for the integration of diagnosis and treatment (Liu 2021; Roy et al. 2023). For example, drug-loaded probes can accurately release anti-inflammatory or lipid-lowering drugs under imaging guidance, or directly ablate plaques through photothermal/photodynamic effects to promote personalized treatment. In addition, fluorescent probes have advantages in revealing pathological mechanisms such as oxidative stress and calcification. The low cost and non-radioactive nature of these materials (generally synthesized from small molecules and organic matter) further accelerate the transition from basic research to clinical translation (Gao 2021; Seah et al. 2023). The multifaceted advantages of the above fluorescent probes make them a powerful tool for early detection and precise therapeutic and therapeutic monitoring of AS, although they also face the challenges of limited tissue penetration and photostability (Wei 2023; Jia et al. 2024).

Fluorescent probes can be divided into two categories: organic fluorescent probes and inorganic fluorescent probes (Wang et al. 2022; Zhang et al. 2022). Organic fluorescent probes are based on conjugated organic compounds that emit fluorescence via radiative transitions following photoexcitation. These probes usually contain conjugated double-bond systems, enabling electron excitation from the ground state to the excited state upon illumination, followed by a radiative return to the ground state with fluorescence emission. In contrast, inorganic fluorescent probes are based mainly on the fluorescence characteristics of inorganic compounds (Kagan et al. 2016). Quantum dots are typical inorganic fluorescent probes composed of semiconductor nanocrystals. Organic fluorescent probes have the advantages of strong and sensitive fluorescence, good optical stability, easy structural modification, good biocompatibility, and small molecular size. However, they also face limitations, including aggregation-induced quenching, susceptibility to photobleaching, limited performance in single-photon imaging, and suboptimal specificity (Wang and Tang 2019; Ma 2021; Hu et al. 2021). Inorganic fluorescent probes are characterized by strong light stability, long fluorescence lifetimes, high quantum yields, wide emission wavelength ranges, and tunable sizes. However, they have poor biocompatibility, difficult metabolism in organisms, weak specific binding ability, and difficult synthesis (Wang 2024; Zhu et al. 2024). Therefore, the selection of fluorescent probes must be tailored to specific application scenarios to maximize their efficacy.

Nowadays, with the wide application of fluorescent probes, diverse arrays of probes that target different cells and tissues have emerged. In this context, the present review aims to summarize the research mechanism and recent progress in the classification, synthesis, targeting strategies, and diagnostic and therapeutic application of fluorescent probes in the field of AS. These efforts are intended to support further development and facilitate the advancement of these technologies.

## 2. Classification and synthesis of fluorescent probes

Fluorescent probes have become essential tools in medical diagnostics and biomedical research owing to their distinctive advantages: exceptional sensitivity and specificity, real-time dynamic monitoring capabilities, non-invasive detection features, and high-resolution imaging at cellular/molecular levels (Gil et al. 2021; Zhang et al. 2023). Contemporary classification systems recognize two primary categories of fluorescent probes: organic-based systems (e.g. small-molecule fluorophores) and inorganic nanomaterials. The synthesis methodologies primarily encompass chemical synthesis, biosynthesis, and physical preparation techniques (Ni et al. 2016; Duan et al. 2023). During practical implementation, critical parameters, including photostability, biocompatibility, and target selectivity, require tailored synthetic approaches to optimize probe performance for specific diagnostic applications. This adaptive design philosophy ensures that each probe system achieves optimal functionality across diverse clinical scenarios.

### 2.1. Classification of fluorescent probes

Fluorescent probes are conventionally categorized based on three principal criteria: chemical architecture, response mechanisms, and application domains. Chemical structures can be divided into two major categories based on the synthetic materials of the probes: organic and inorganic. The response mechanism can be classified into physical and chemical response based on the target components it detects. The application domains are mainly reflected in vivo imaging and in vitro component detection.

The organic/inorganic classification system distinguishes: (1) organic probes (e.g. fluoresceins, rhodamine derivatives, GFP variants), which are characterized by structural tunability and superior biocompatibility; (2) inorganic probes (e.g. quantum dots, rare-earth-doped nanomaterials, metal nanoclusters), which exhibit enhanced photostability and precisely tunable emission profiles. This functional complementarity enables their synergistic deployment across biomedical imaging and environmental monitoring applications.

#### 2.1.1. Organic fluorescent probes

Current classification frameworks for organic fluorescent probes primarily recognize four structural families: fluoresceins, rhodamines, cyanines, and coumarins. Fluorescein derivatives characterized by benzopyran-based conjugated systems. Botezatu et al. (2023) exhibit exceptional aqueous solubility and high quantum yields (Feng et al. 2023; Moon et al. 2023; Wang et al. 2024), with molecular engineering strategies enabling precise modulation of emission profiles through substituent modifications, (Huang 2023) as demonstrated in fluorescein isothiocyanate (FITC) biomolecular labeling applications (Sato et al. 2014). Rhodamine analogues featuring xanthene ring architectures (Jiang 2021) maintain comparable quantum yields while demonstrating superior photobleaching resistance (Grimm 2023; Kim 2023), with amino group functionalization producing derivatives such as tetramethylrhodamine that span 550–650 nm emission ranges for multiplexed cellular imaging (Bucevičius 2023; Zhang 2023). Cyanine systems, distinguished by nitrogenous heterocycles interconnected via polymethine bridges, (Aparin 2022; Xu 2022; Li et al. 2023) achieve near-infrared (NIR) emission (650–900 nm) with large Stokes shifts, a critical feature exploited in indocyanine green derivatives for deep-tissue visualization (Usama et al. 2021; Fukushima 2022; Wilson and Sletten 2023). The coumarin-based fluorescent probe exhibits excellent photostability and a high fluorescence quantum yield. Through rational modification of substituent groups in its molecular framework, precise tuning of the fluorescence emission wavelength can be achieved (Cui 2021; Liu et al. 2023; Liu et al. 2023). This class of probes has found extensive applications in diverse analytical fields, including biosensor development, pharmaceutical analysis, and bioanalytical chemistry. Specifically, it enables sensitive and selective detection of various analytes, such as metal ions, biomolecules, and other target species in complex matrices (Zhang et al. 2022; Olowolagba 2024).

#### 2.1.2. Inorganic fluorescent probes

Inorganic fluorescent probes are conventionally classified into three principal categories: quantum dots (QDs) systems, rare-earth-doped materials, and metal nanoclusters. QD probes consist of semiconductor nanocrystals with size-tunable optoelectronic properties (Zhang 2021; Park 2022). The fluorescence emission wavelength of QDs can be tuned by altering their size, composition, and structural architecture (Ruiyi et al. 2022). These nanomaterials feature a broad excitation spectrum, narrow symmetrical emission profiles, high fluorescence quantum yields, and robust photostability. Their versatile applications span biological imaging, biosensor development, and related fields, with representative examples including CdSe, CdS, and analogous quantum dot systems (Sanjayan and Geetha Balakrishna 2023). Meanwhile, the development of carbon quantum dots (CQDs) has demonstrated remarkable potential in fluorescent probe applications (Jiang et al. 2023). Additionally, leveraging the unique electronic shell structures and optical properties of rare earth elements, researchers have developed rare earth-doped inorganic fluorescent probes via the doping of rare earth ions into various inorganic matrices (Zhong 2017; Qu 2020; Song et al. 2022). Characterized by long fluorescence lifetimes, narrow emission peaks, and large Stokes shifts, these probes exhibit significant applications in fields such as biological detection and optical anti-counterfeiting (Ding 2022). Prominent examples of rare earth ion-doped fluorescent probes include Eu³⁺, Tb³⁺, and other lanthanide ion systems (Guo et al. 2021). Metal nanocluster-based probes typically consist of several to dozens of metal atoms, with sizes on the order of the electron Fermi wavelength, exhibiting atomic-like discrete electronic states and molecule-like fluorescence properties (Jia et al. 2012; Song 2024). The fluorescence emission wavelength of these probes can be precisely tuned by adjusting the composition, size, and ligand environment of the nanoclusters (Xiao et al. 2015; Ran et al. 2021; Xia 2025). Characterized by excellent biocompatibility and water solubility, have significant potential in biomedical applications, including but not limited to Au and Ag nanoclusters (Xiao et al. 2014; Feng 2018).

### 2.2. Synthesis of fluorescent probes

#### 2.2.1. Chemical synthesis

The chemical synthesis of fluorescent probes is fundamentally rooted in organic synthetic chemistry principles, where precise engineering of reaction pathways enables covalent linkages between fluorophores and recognition units to construct target-specific probe architectures. This approach achieves precise modulation of optical properties (e.g. emission wavelength, quantum yield) and recognition selectivity through systematic molecular design, though requiring sophisticated multi-step syntheses with rigorous purification protocols. Current methodologies predominantly employ seven reaction mechanisms: (1) The condensation reaction method, in which covalent bonds are formed by condensation between active functional groups (Wang et al. 2017; Huang 2020; Sadia 2023; Xu et al. 2023). For example, the fluorescent product is formed by a Schiff base reaction (condensation of the aldehyde group and hydrazine group), and the condensation reaction between active functional groups turns on the signal (He and Song 2022). (2) The redox reaction method regulates the molecular conjugated system through electron transfer (Kaur et al. 2015; Lou et al. 2015; Belzile et al. 2016; Zhang et al. 2024). An NIR two-photon fluorescent probe was prepared by regulating the conjugated structure through the redox reaction initiated by hypochlorous acid (Luo et al. 2025). (3) The substitution reaction method introduces recognition sites by functional group substitution (Işık 2014; Gong 2017). For example, the amino group of 4-CF_3_-7-aminoquinoline as a nucleophile reacts with the active halogenated hydrocarbon group of 2-crotonyloxymethyl–2-cyclohexenone (such as chloromethyl) to form a new C–N bond, thereby connecting the two to construct a probe molecule (Rong 2021). (4) Coordination reaction method using metal‒ligand complexation (Li 2021; Li et al. 2022; Wang 2023; Sahu 2025), such as thiosemicarbazide ligand L through the N/S atoms and Hg^2+^ coordination, to achieve detection (Mohanty 2023). (5) The addition reaction method introduces a response group at the double bond site through unsaturated bond functional modification (Liu et al. 2012; Liu et al. 2016; Wan 2021; Yang et al. 2023). (6) The fluorescent unit is embedded in the polymer skeleton by a polymerization reaction, and the polymer probe material is prepared by free radical polymerization and other strategies (Madsen et al. 2011; Yamada et al. 2015; Zhang et al. 2018; Tahseen et al. 2022). These methods have different emphases, among which condensation and substitution reactions are more suitable for the construction of small-molecule probes, while self-assembly and polymerization methods are conducive to the development of multifunctional composite probe systems. Although the chemical synthesis method has the challenges of a complex process and limited yield, the flexibility of molecular design and the adjustability of performance make it maintain an important position in the fields of biosensors and environmental monitoring.

#### 2.2.2. Biosynthesis method

The biosynthesis of fluorescent probes is accomplished by utilizing enzymatic reactions, metabolic pathways, and genetic regulatory mechanisms within biological systems to construct such probes. Its significant advantage lies in the high biocompatibility and targeting specificity of the product, but it relies on complex bioengineering technology support. At present, the main strategies include the following five categories. (1) The enzymatic reaction method relies on biocatalysts such as oxidoreductases to directionally convert precursors (Bouldin et al. 2014; Nakamura et al. 2019; Vallejo et al. 2019; Tsarkova 2021; Zhong 2021; Botezatu et al. 2023), such as fluorescein, under mild conditions, such as in situ synthesis of probes by laccase-catalyzed oxidation of fluorescent substrates. (2) The microbial fermentation method uses the metabolic pathway of engineered strains to produce fluorescent molecules on a large scale (Cui 2009; Li 2020; Schmitz and Rosenbaum 2020; Dramicanin et al. 2023). For example, Pseudomonas fluorescens synthesizes phenazine fluorescein through the polyketide synthase pathway, and the yield can be improved to an industrial level by optimizing the fermentation process. (3) The endogenous metabolic approach enables in situ monitoring of mitochondrial oxidative stress by regulating the activity of metabolic enzymes (e.g. cytochrome P450), thereby inducing fluorescence responses of intracellular lutein-like substances (Brenner et al. 2008; Walsh et al. 2021; MüLLEROVá et al. 2022). (4) The genetic engineering method uses recombinant DNA technology to express fluorescent protein probes (Shahravan et al. 2011; Ali et al. 2018; Chen et al. 2023). Typically, the calcium-sensitive GCaMP6f gene circuit is introduced into neurons to achieve dynamic imaging of neural electrical activity (Cichon 2020). (5) The cell culture method produces green fluorescent protein GFP in tobacco cells by genetic engineering, cell secretion pathway regulation, protein folding, and function maintenance (Su et al. 2004). Compared with traditional chemical synthesis, biosynthetic systems have outstanding in vivo compatibility. Among these methods, genetic engineering and enzymatic methods have advantages in terms of molecular design accuracy, while microbial fermentation and cell culture methods are more suitable for large-scale production. Despite the challenges of complex metabolic regulation and difficult product separation, these methods have shown unique application value in biomedical fields, such as in vivo imaging and diagnosis and treatment integration.

#### 2.2.3. Physical synthesis method

**Figure 1.**
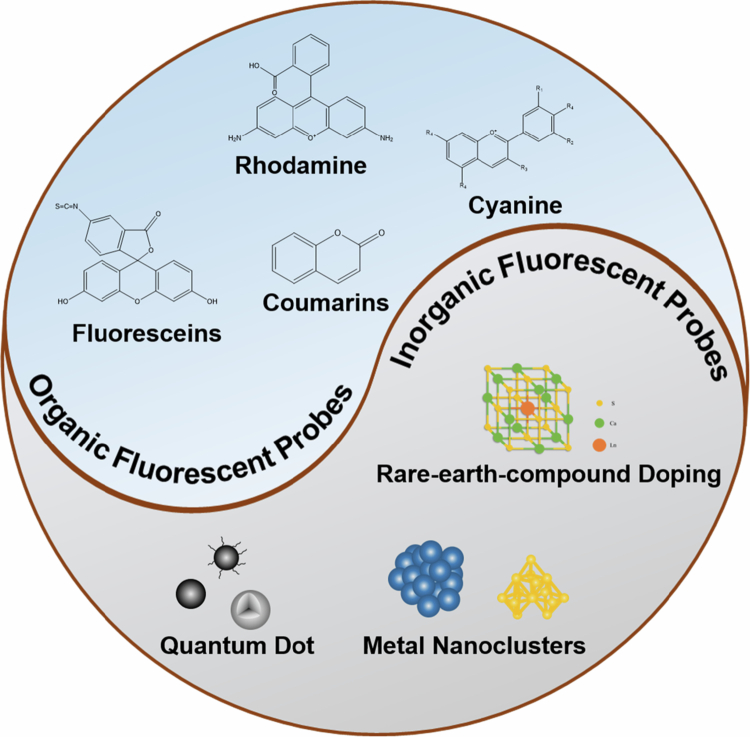
Classification of fluorescent probes.

The physical synthesis method of fluorescent probes uses mainly the non-chemical bonding mechanism driven by the energy field to realize the physical coupling of fluorophores and recognition groups. Its core feature is to avoid complex organic reaction steps, but there is an inherent limitation of insufficient molecular structure accuracy. The current physical synthesis strategy mainly covers the following five types of technical systems: (1) Physical vapor deposition of metal oxide fluorescent films (such as ZnO fluorescent sensing films) on the surface of the substrate by evaporating precursors such as metal halides at high temperature (Fédéli et al. 2015; Mandal et al. 2021; Nur-E-Alam 2023). Crystallinity can be controlled by the deposition rate and substrate temperature. (2) CQDs were synthesized via the arc discharge method using high-voltage arc instantaneous evaporation of the carbon source (Singh et al. 2018; Gayen et al. 2019; Chao-Mujica 2021). Although the product has an excellent fluorescence quantum yield (approximately 45%), it is limited by particle size polydispersity (2−8 nm) and low yield ( < 30%). (3) CQDs were prepared via the laser ablation method using a high-energy pulsed laser (such as Nd:YAG, 1064 nm)-vaporized graphite target (Thongpool et al. 2013; Dudek 2017). Monodisperse nanoparticles (PDI < 0.15) can be obtained, but the equipment cost is high, and the quantum yield is only 20%–25%. (4) The RF discharge method dissociates CH_4_ plasma in a 13.56 MHz RF field to synthesize uniform-sized CQDs (3.5 ± 0.7 nm) (Ma et al. 2019; Lisovskiy et al. 2022; Mohammadzaheri et al. 2022). Its continuous production mode increases the yield to more than 60%, but it needs to be equipped with a vacuum system and an exhaust gas treatment device. (5) The ultrasonic treatment method mechanically exfoliates carbon materials to prepare CQDs through the cavitation effect (20 kHz, 500 W), which has the advantage of green synthesis, but the fluorescence intensity of the product is usually lower than that of the chemical synthesis system (quantum yield is approximately 12%) (Dang et al. 2016; Kastilani et al. 2019; Kim et al. 2019; Azam et al. 2021). Among these methods, vapor deposition technology is more suitable for the construction of rigid fluorescent film materials, while the discharge method and the laser method dominate the preparation of carbon-based quantum dots. Although physical methods generally face challenges such as uncontrollable surface functional groups and random distribution of recognition sites, their process simplicity and solvent-free characteristics have shown unique value in the development of environmentally sensitive probes, especially in the field of in vivo imaging. The low cytotoxicity advantage of physically synthesized CQDs has been verified by many in vivo experiments (Figure 1a).

## 3. Application of fluorescent probe technology in the study of atherosclerosis mechanism

The progression of AS is accompanied by a cascade of biological events, including lipid accumulation, increased reactive oxygen species, the release of inflammatory factors, the phosphorylation of proteins, and other unidentified molecular mechanisms. Existing fluorescent probes targeting AS are predominantly developed against these biomolecular events (Table 1). Targeting key molecular intermediates in AS pathogenesis enables systematic elucidation of its developmental trajectory and facilitates therapeutic intervention by disrupting these pathogenic pathways.

**Table 1.** Targeting sites and mechanism of fluorescent probes for atherosclerosis.

Author	Fluorescent probe materials	Fluorescent wavelength	Targets	Basic action
Jiandong Liu (Liu 2024)	TPA, pyridine, methyl cinnamate	λ_ex_ = 405 nm λ_em_ = 420−460 nm	LDs	Water repellent
Di Ma (Ma et al. 2023)	TPE derivatives	λ_ex_ = 481 nm λ_em_ = 643 nm	LDs	Water repellent AIE
Jingkang Li (Li et al. 2025)	Nile red derivatives	λ_ex_ = 580 nm λ_em_ = 690 nm	CHE, LDs	Enzymatic response
Yan Huang (Huang 2022)	Cy-NOH2	λ_ex_ = 605 nm λ_em_ = 750 nm	H_2_O_2_	ROS activation
Qiaolei Wang (Wang et al. 2021)	Naphthalimides	λ_ex_ = 411 nm λ_em_ = 486 nm	·OH	ROS activation
Boxuan Ma (Ma et al. 2021)	Carbazole, indene diketone	λ_ex_ = 401 nm λ_em_ = 580 nm	ROS, LDs	ROS dissociation
Jie Liu (Liu 2023)	Triphenylamines	λ_ex_ = 488 nm λ_em_ = 650 nm	PS, LDs	ROS activation
Lin Shen (Shen 2024)	BBT-2FT	λ_ex_ = 808 nm	VCAM−1, M1, M2	Receptor ligand combination
Qiuyu Gong (Gong et al. 2018)	Half-cyan	λ_ex_ = 670 nm λ_em_ = 700 nm	PGP−1	Enzymolysis, off-on
Lin Zou (Zou 2023)	Cy7-NHS	–	CCR2, F4/80	Initiative + passive
Xiaoxia Li (Li 2019)	–	–	e-Selectin	Targeted enrichment
Yin Shi (Shi 2024)	–	–	Integrin αvβ3	Targeted
Siting Zhang (Zhang 2025)	–	–	Ceramide	Targeted

### 3.1. Fluorescent probes target lipid deposition in atherosclerosis

**Figure 2.**
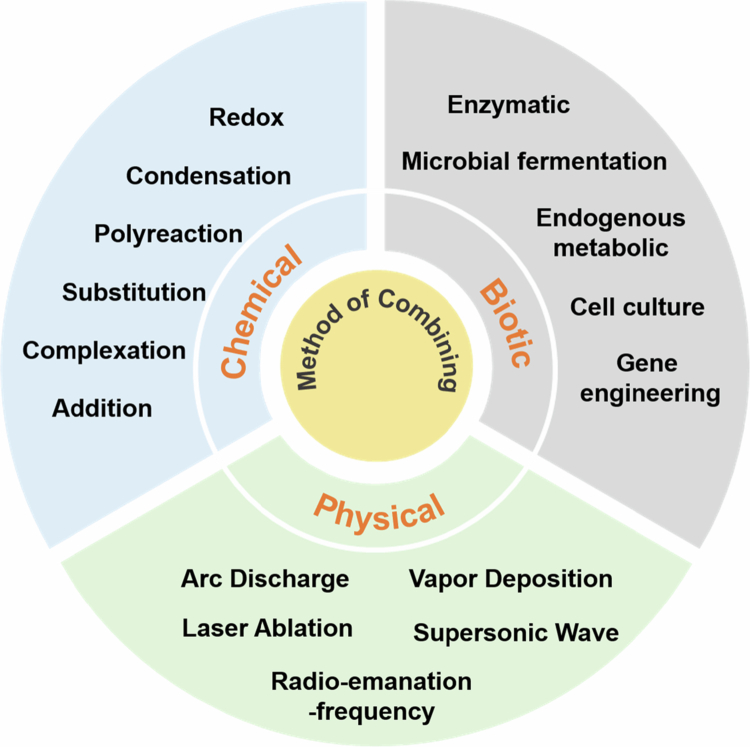
Synthesis method of fluorescent probe.

The design of fluorescent probes for imaging abnormal lipid metabolism is grounded in the specific molecular recognition mechanisms between lipid molecules and biological macromolecules within the pathological microenvironment. The core design strategy entails the synergistic integration of molecular recognition elements, microenvironment response modules, and signal amplification systems. Through the specific binding of molecular recognition elements (e.g. apolipoproteins, oxidized low-density lipoprotein antibodies, or aptamers) to target-specific antigen‒antibody complexes or ligand‒receptor pairs, the probe enables spatially selective accumulation in lipid deposition regions (Figure 2).

**Figure 3.**
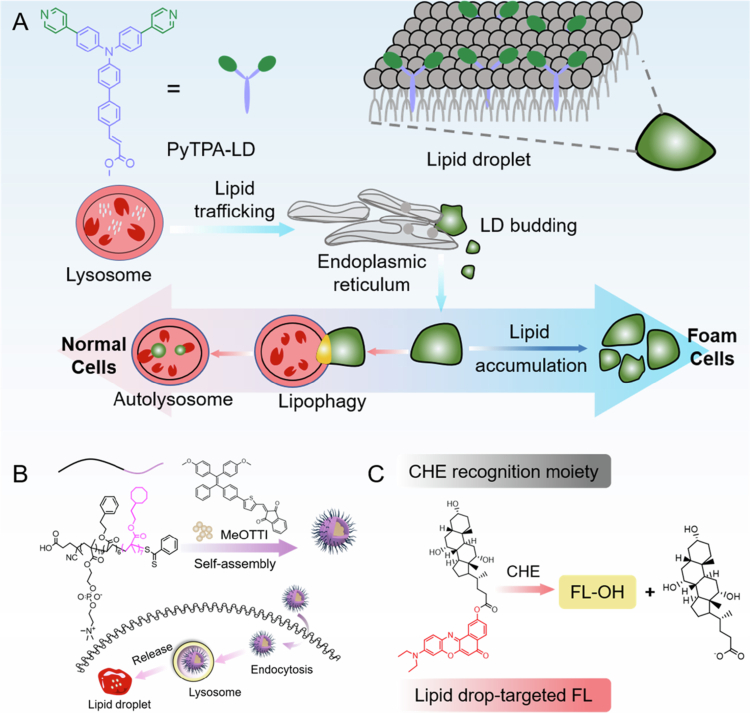
(a) The chemical structure of PyTPA-LD and its targeting mechanism toward lipid droplets (Liu 2024). (b) LDs-specific imaging of MeOTTI-PMEA NPs in cells and atherosclerotic plaques (Ma et al. 2023). (c) The enzymatic response mechanism of the probe (Li et al. 2025).

Liu et al. reported the triphenylamine skeleton probe PyTPA-LD, which optimized the targeting performance via the hydrophilic‒hydrophobic dynamic balance of its molecular structure: the pyridine group binds to phospholipid heads through electrostatic interactions, the methyl cinnamate side chain embeds into the hydrophobic core of lipid droplets, and the D-*π*-A electron transport system cooperates with the intramolecular charge transfer mechanism to produce a polarity-dependent fluorescence enhancement effect (Figure 3a) (Liu 2024). After 50 laser scans in A549 and RAW264.7 cells, the probe retained >90% of its fluorescence intensity. Its colocalization coefficient with Nile red and commercial BODIPY dyes reached 0.83 and 0.75, respectively, confirming its excellent photostability and targeting specificity. In an ox-LDL-induced macrophage foam cell model, PyTPA-LD dynamically revealed that lysosomes promoted lipid droplet formation via ox-LDL degradation, whereas starvation or rapamycin-activated lipophagy significantly removed lipid droplets. Furthermore, in a high-fat diet–fed *ApoE*^-/-^ mouse atherosclerosis model, the spatial distribution of probe-labeled lipid droplets was highly consistent with the oil red O staining results (*R*^2^ = 0.91), indicating successful in situ visualization of lipid deposition in pathological tissues. This system overcomes the rapid photobleaching defect of traditional dyes and, for the first time enables 24 h continuous tracking of dynamic lipid droplet–lysosome interactions, providing a high spatiotemporal resolution research tool for analyzing the mechanisms of lipid metabolism imbalance in AS.

In the field of probe molecular engineering, the design of the hydrophobic moiety directly influences the probe’s positioning accuracy and retention performance. Ma et al. synthesized a MeOTTI probe via a Suzuki coupling reaction, employing 4,4'-(2-(4-bromophenyl)-2-phenyl-1,1-diyl) dimethoxybenzene as the molecular backbone (5-formylthiophene-2-yl) boric acid as the functional monomer, which was then condensed with 1,3-indolinone under the catalysis of CsF and Pd (t-Bu_3_*P*)_2_ to construct a tetraphenylethylene (TPE) electron-donating unit (Figure 3b) (Ma et al. 2023). The probe exhibits an absorption peak at 472 nm and an emission peak at 655 nm in the aqueous phase, with a Stokes shift of 183 nm, and displays typical aggregation-induced emission (AIE) characteristics: when the volume fraction of water in the water/DMSO mixed solvent increases from 0% to 70%, the fluorescence intensity increases by 6.8 times, confirming that restricted molecular motion effectively suppresses non-radiative transition. Following electrostatic self-assembly loading of MeOTTI onto the core-shell nanoparticles formed by poly(methyl methacrylate-coethylene glycol-acrylamide, PMEA), the probe’s hydrophobicity significantly improved, with the particle size increasing from 98.3 nm (PMEA) to 132.7 nm (MeOTTI-PMEA NPs) while maintaining colloidal stability in PBS for 72 h. In co-localization, the Pearson coefficient between MeOTTI and lysosomes decreased from 49.58% to 26.13%, and the coefficient between MeOTTI and LDs increased from 70.25% to 91.57% after incubation for 1−6 h. After the NPs were endocytosed into the cells, MeOTTI was released in the acidic environment of the lysosomes and targeted LDs. Notably, in a pH 6.8 microenvironment, protonation of the nitrogen atom in the acrylamide group in the PMEA chain increases the zeta potential of the nanoparticles from −23.1 mV to +15.4 mV, leading to structural disassembly and accelerated probe release. This feature provides a molecular basis for specific imaging of atherosclerotic acidic plaques. *ApoE*^-/-^ mice fed a high-fat diet for 14 weeks were injected with 200  μL of MeOTTI-PMEA NPs (7  mg/mL) via the tail vein and sacrificed after 24  h. The aortic plaques in the experimental group showed strong fluorescence.

Enzyme-responsive probes achieve precise signal regulation through molecular conformational transformation, offering unique advantages in metabolic dynamic monitoring. Li et al. developed the near-infrared (NIR) probe NR-CHE, which incorporates cholic acid as the recognition moiety, linking the Nile red fluorophore to a cholesterol esterase (CHE)-sensitive substrate via an ester bond (Figure 3c) (Li et al. 2025). The probe specifically undergoes hydrolysis catalyzed by CHE, with the released Nile red molecule exhibiting a 12.3-fold increase in fluorescence intensity upon lipid droplets. It has a detection limit of 0.076 U/mL (linear range 0−0.6 U/mL) and a cross-reaction rate of <5% with 32 bio-interfering substances (including reactive oxygen species, proteases, etc.). Molecular docking analysis revealed that NR-CHE establishes a hydrogen bond network with the active center of CHE through the ARG-63 and TYR-103 residues, with a binding energy of 9.9 kcal/mol, confirming its high affinity characteristics. In an ox-LDL-induced foam cell model, the probe fluorescence intensity is 3.2-fold higher than that in normal macrophages, with a colocalization coefficient of 0.977 with BODIPY dye. Animal experiments showed that the fluorescence signal intensity at the arterial plaque of *ApoE*^-/-^ mice was positively correlated with the pathological process (*r *= 0.89). When extended to a liver cancer model, the probe enables accurate tumor boundary definition (signal-to-noise ratio > 8.6) and improves the accuracy of guiding surgical resection to 93.4%.

However, owing to the common defect of non-unique targets in probes, the current research trend focuses on the construction of multifunctional nanocarriers and the integration of multimodal imaging technologies. Current research efforts increasingly focus on constructing multifunctional nanocarriers and integrating multimodal imaging technologies. For instance, hyaluronic acid-functionalized liposomes enable CD44 ligand-mediated active targeting of plaque macrophages (Lee et al. 2015), and by integrating Raman and fluorescence dual-mode imaging, spatial resolution can be achieved at the subcellular level ( < 200 nm). These technological breakthroughs not only enable dynamic tracing of lipid metabolism in atherosclerotic plaques but also provide a molecular visualization platform for elucidating the mechanisms of lipid‒inflammation‒fibrosis cascade reactions and developing targeted therapeutic strategies.

### 3.2. Specific recognition of reactive oxygen species in the process of atherosclerosis by fluorescent probes

The design of fluorescent probes targeting ROS adheres to the principle of close integration between molecular engineering and the characteristics of the pathological microenvironment, with the core mechanism of such probes centering on the precise modulation of specific recognition elements and signal transduction modules. A probe system based on oxidation-sensitive chemical bonds (thioether bonds, borate ester bonds) triggers the activation or enhancement of fluorescence signals through oxidative cleavage to achieve dynamic monitoring of ROS.

**Figure 4.**
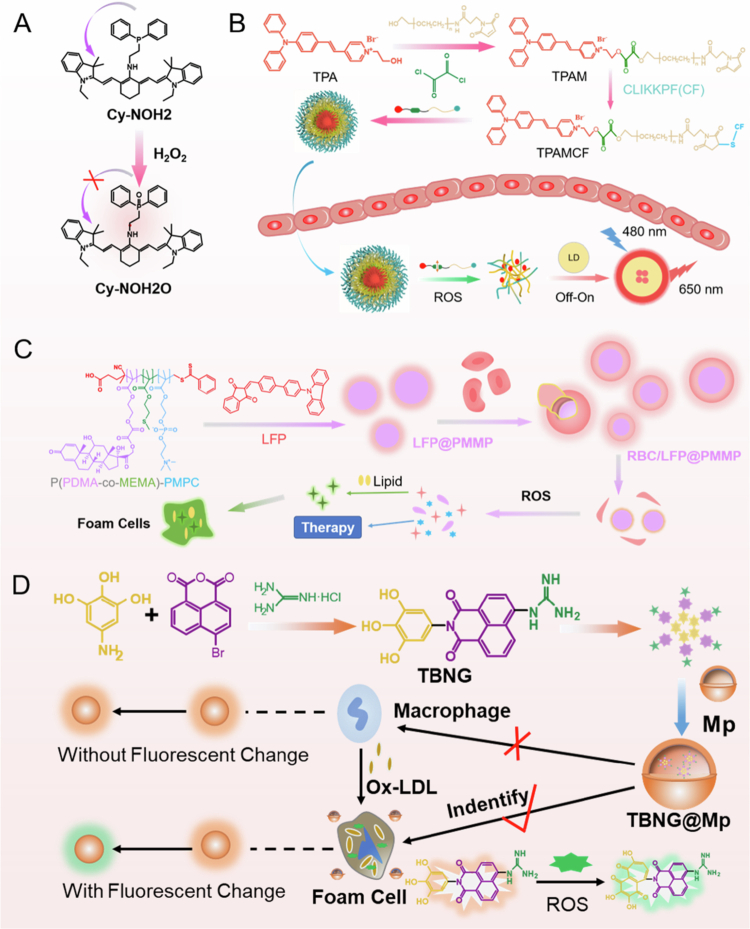
(a) The detection mechanism of probe Cy-NOH2 for H_2_O_2_ (Huang 2022). (b) A schematic diagram of Ros-triggered nanoparticle disassembly and AIE imaging of Tpamcf NPS (Liu 2023). (c) A schematic diagram of RBC/LFP@PMMP formation and its response mechanism to ROS and lipid droplets (Ma et al. 2021). (d) The schematic diagram of the synthesis and response mechanism of the nano-detection system TBNG@Mp based on naphthalimide (Wang et al. 2021).

Huang's research team synthesized Mg (DHT) metal‒organic framework probes via a solvothermal method (Figure 4a) (Huang 2022). Using Mg (NO_3_)_2_·6H_2_O, 2,5-dihydroxyterephthalic acid (DHT), and triethylamine as precursors, highly crystalline yellow crystals were successfully prepared. Single-crystal X-ray diffraction analysis revealed that the Mg^2+^ center forms an octahedral coordination configuration with DHT ligands and DMF molecules and maintains excellent structural stability after desolvation. The aqueous dispersion of the probe exhibited strong fluorescence emission at 556 nm under 365 nm excitation, with a Stokes shift reaching 191 nm, demonstrating superior photostability and aqueous dispersibility. Under the action of hydroxyl radicals (·OH) and hypochlorite (ClO-), the probe’s fluorescence intensity was quenched in a concentration-dependent manner. UV-visible absorption and Fourier transform infrared spectroscopy confirmed that hROS induced the oxidation of the hydroquinone moiety to a quinone structure, disrupting the intramolecular hydrogen bond network and inhibiting the excited-state intramolecular proton transfer (ESIPT) process. LO2 cells were treated with bisphenol A (BPA) at concentrations of 0, 5, 10, and 20 μM for 24 h, followed by incubation with 10  μM Cy-NOH₂ for 40  min. After that, the fluorescence intensity increased significantly with increasing BPA concentration. The results from the H₂O₂ detection kit were consistent with the imaging results, confirming that BPA induced H₂O₂ accumulation in a dose-dependent manner. Meanwhile, detection at different induction time points (0–24 h) confirmed its time dependence. For in vivo mouse experiments, 6–8-week-old BALB/c mice (*n* = 20) were used. The groups used were as follows: the control group (0 μg/kg), low-dose group (0.4 μg/kg), medium-dose group (4 μg/kg), and high-dose group (40 μg/kg), with oral solution treatment applied. The higher the BPA concentration was, the stronger the overall fluorescence signal in the mice was, which proved that the in vivo H₂O₂ content increased with increasing BPA concentration. This study reports the first MOF fluorescent probe based on redox-reversible ESIPT mechanism, featuring a large Stokes shift (191 nm), high sensitivity (detection limit of 0.21 μM), excellent biocompatibility, and reversible response characteristics, thereby providing a novel molecular tool for dynamic tracing of endogenous/exogenous hROS in living cells.

In the field of molecular probe design, redox-responsive fluorophores (such as phenylboronic acid groups, selenocysteine) modulate their responses to ROS dynamics via electron transfer mechanisms or conjugated system reconfiguration. Wang et al. constructed a bifunctional probe system comprising TBNG and GNTB through multi-step condensation reaction: using 3,4,5-trihydroxybenzamide, 4-bromo-1,8-naphthalic anhydride, and guanidine hydrochloride as raw materials, the target product was synthesized via nucleophilic substitution and amidation reaction (Figure 4) (Wang et al. 2021). The spectral characterization revealed that the TBNG probe exhibited a maximum UV absorption at 347 nm and a maximum emission peak at 486 nm under a 411 nm excitation wavelength. In oxidation mechanism studies, ROS can specifically oxidize the gallic acid-derived hydroxyl moiety to generate quinone derivatives, resulting in a change in the *π*-π*electronic transition energy level of the naphthalimide conjugate system and a corresponding redshift in fluorescence emission (*λ* = 39 nm). Hydroxyl radical scavenging experiments confirmed that the scavenging efficiency of TBNG and GNTB for ·OH reached 68.3 ± 2.7% and 72.1 ± 3.1%, respectively, significantly outperforming the positive control vitamin C (58.9 ± 1.8%). The synergistic integration of oxidative scavenging and fluorescence response provides a dual mechanism for the specific recognition of the probe in complex biological systems. In the biotoxicity experiments, the toxicity of free TBNG/GNTB increased with increasing concentration, while the toxicity of modified TBNG@Mp/GNTB@Mp significantly decreased. Among them, TBNG@Mp exhibited the lowest toxicity – at a concentration of 120  μg/mL, the survival rate of HUVECs was still >85%, without damaging the vascular endothelium. Two hours after injecting TBNG@Mp into rats with early-stage AS, obvious fluorescent signals were observed in the thoracic aorta, while no fluorescent signals were detected in normal rats. These findings prove that TBNG@Mp can be used for the early detection of AS.

To increase probe lesion-targeting efficiency, researchers have developed diverse active delivery systems. The Ma team constructed an erythrocyte membrane-derived biomimetic nanotheranostic platform (RBC/LFP@PMMP) that integrates a lipid-responsive fluorescent probe (LFP) and an ROS-sensitive prodrug delivery system (PMMP) (Figure 4c) (Ma et al. 2021). The platform has the following innovative features: (1) The LFP probe achieves lipid environment-specific fluorescence conversion through an intramolecular torsion mechanism (lipid-rich region 525–580 nm). (2) The PMMP copolymer loaded with the prednisolone prodrug achieved controlled release in the ROS-overexpressing region (72 h cumulative release rate was 83.6%). (3) Erythrocyte membrane camouflage extended the circulation half-life to 4.3 times that of bare nanoparticles, and the plaque-targeting efficiency in the *ApoE*^-/-^ mouse AS model was increased by 2.1-fold. Treatment experiments demonstrated that the platform reduces the plaque area by 60.2 ± 4.8% and significantly downregulates the inflammatory factors TNF-*α* and IL-1β, achieving theranostic integration of visual positioning and precise treatment.

In the domain of signal-specific regulation, the microenvironment logic gating strategy demonstrates distinct advantages. The TPAMCF nanoprobe system developed by Liu et al. is based on a dual pH/ROS-responsive mechanism (Figure 4b) (Liu 2023). Specifically, an aggregation-induced emission (AIE) fluorescent core is constructed via the Knoevenagel condensation reaction between 4-(diphenylamino)benzaldehyde and malononitrile. A targeting peptide (CF peptide) is conjugated to this core through an ROS-sensitive linker, forming an intelligent probe. This system has three regulatory features: it remains in a fluorescent silent state (off) under physiological pH conditions, activates targeted recognition within the atherosclerotic microenvironment (pH 6.5–6.8), and triggers fluorescence recovery (on) along with the release of therapeutic molecules in response to ROS. HeLa cells and RAW 264.7 cells were treated with 1 mg/mL TPAMCF NPs for 24 h. The cell viability detected by the MTS assay was > 90%, with no obvious toxicity. For the animal experiments, 8-week-old male *ApoE*^*⁻/⁻*^ mice were fed a high-fat diet for 8 weeks to establish the AS model. These mice were then given tail vein injections of TPAMCF NPs, TPAM NPs, or normal saline and sacrificed at 6, 12, and 24 h, respectively, to detect fluorescence in the aorta and major organs. The aortic fluorescence intensity in the TPAMCF group was significantly higher than that in the TPAM group (*P* < 0.05) and reached a peak at 12 h. The fluorescent signal regions completely matched the plaque locations identified by Oil Red O staining, which proves that TPAMCF NPs can label plaques accurately. The in vivo imaging results indicate that the fluorescence intensity of the probe at aortic plaques is 8.7-fold higher than that in normal vascular tissue, resulting in a signal-to-noise ratio of 23.6:1. This performance significantly surpasses that of traditional probe systems (*P* < 0.01).

These advanced probe systems not only provide tools with high spatiotemporal resolution for the dynamic visualization of oxidative stress in AS but also establish a novel paradigm for investigating the kinetics of disease progression and evaluating therapeutic via multimodal signal feedback mechanisms. Their design concept provides an extensible technical framework for the molecular diagnosis and treatment of other oxidative stress-related diseases.

### 3.3. Specific recognition of fluorescent probes to inflammatory factors

The design of fluorescent probes that target atherosclerotic inflammatory factors is focused on the dynamic recognition of pro-inflammatory mediators (e.g. TNF-*α*, IL-6, and IL-1β) and their signal transduction in the pathological process. The core strategy involves accurately targeting the lesion area through specific binding to inflammatory factors or cell surface markers (e.g. macrophage CD68 and endothelial cell ICAM-1) through molecular recognition elements (such as monoclonal antibodies, aptamers, or chemokine receptor ligands).

**Figure 5.**
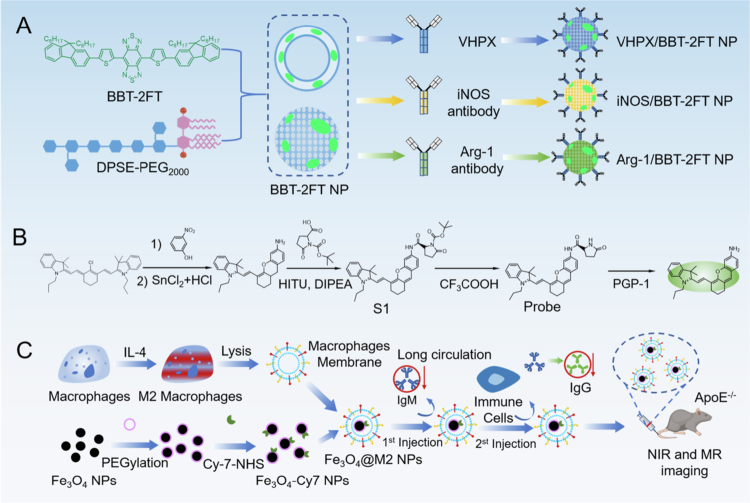
(a) The chemical structure of BBT-2FT and the synthesis schematic diagram of VHPK/BBT-2FTNPs, iNOS/BBT-2FTNPs, Arg-1/BBT-2FTNPs (Shen 2024). (b) The probe synthesis and its reaction with PGP-1 (Gong et al. 2018). (c) The synthesis schematic diagram of Fe_3_O_4_@M2 NPs probe (Zou 2023).

For example, Shen et al. synthesized VHPK/BBT-2FTNPs, iNOS/BBT-2FTNPs, and Arg-1/BBT-2FTNPs probes using a thin-film hydration method to incorporate the VHPK peptide, iNOS antibody, and Arg-1 antibody into BBT-2FT liposomes (Figure 5a) (Shen 2024). These four liposome probes exhibited uniform particle size, similar negative zeta potentials, good colloidal stability and photostability, and no apparent cellular toxicity at high concentrations. VHPK/BBT-2FTNPs showed enhanced uptake in inflamed endothelial cells, while iNOS/BBT-2FTNPs and Arg-1/BBT-2FTNPs demonstrated increased cellular internalization in M1 and M2 macrophages, respectively. In *ApoE*^-/-^ mice, VHPK/BBT-2FTNPs selectively recognized atherosclerotic plaques, whereas iNOS/BBT-2FTNPs and Arg-1/BBT-2FTNPs specifically targeted M1 and M2 macrophages within plaques, respectively, displaying strong fluorescent signals and prolonged retention at lesion sites. Cytotoxicity assay showed that at a high concentration of 1040 μg/mL, the four probes still had no significant effect on the survival rate of bone marrow-derived macrophages. Dynamic monitoring revealed that over 10–40 weeks, the fluorescence intensities of VHPK/BBT-2FTNPs and iNOS/BBT-2FTNPs increased with increasing AS, while that of Arg-1/BBT-2FTNPs decreased. Early-stage plaques were dominated by M2 macrophages, whereas late-stage plaques exhibited a shift toward M1 macrophage dominance. Concurrently, the differential expression of related signaling pathway molecules differed across stages, with the late-stage hypoxic microenvironment promoting M1 macrophage polarization and the inflammatory response, which are correlated with plaque progression and vulnerability rupture.

In probe design, integrated response elements (e.g. MMP-9 cleavable peptide chains for triggering fluorescence release) or microenvironment activation mechanisms (e.g. reactive oxygen species/weak acid synergistic regulation) can dynamically reflect the intensity and spatial distribution of inflammatory signals. For example, Gong et al. synthesized a probe by using a hemicyanine fluorophore as the scaffold, incorporating protected L-pyroglutamic acid, and subsequent deprotection (Figure 5b) (Gong et al. 2018). The probe exhibited an emission wavelength of 670/700 nm, high selectivity for PGP-1 (sensitivity of 0.18 ng/mL), and a linear response over the concentration range of 0.01−0.25 μg/mL. It displayed strong affinity for PGP-1 and good biocompatibility. Following FCA-induced hindlimb inflammation and LPS/D-Gal-induced liver injury in mice, fluorescence imaging revealed enhanced probe signals. Western blot analysis confirmed up-regulation of PGP-1 and tumor necrosis factor-*α* (TNF-*α*), while H&E staining of liver tissues showed inflammation and necrosis, linking PGP-1 to the inflammatory process. This study successfully developed an NIR fluorescent probe, revealing a correlation between PGP-1 upregulation and inflammation under immunopotentiator stimulation. Cellular-level studies demonstrated that PGP-1 inhibition attenuated inflammation, suggesting that PGP-1 may represent a novel inflammatory cytokine.

In addition, probes based on biomimetic delivery platforms (e.g. exosomes or macrophage membrane-coated nanoparticles) can actively home to inflammatory sites and increase detection sensitivity through multimodal imaging (fluorescence/photoacoustic synergy). Zou et al. constructed a dual-modality probe (Fe_3_O_3_@M2 NPs) by coating M2 macrophage membranes onto Fe_3_O_4_-Cy7 nanoparticles via liposome extrusion, enabling high-precision NIR fluorescence and magnetic resonance imaging of AS lesions (Figure 5c) (Zou 2023). The design leveraged the specific binding of the CCR2 protein on the M2 macrophage membrane to the CCL2 ligand in the plaque microenvironment, combined with the T2-weighted imaging capability of the Fe_3_O_4_ core (*r*_2_ relaxation rate ≈ 120 mM^−1^s^−1^) and the NIR fluorescence of the Cy7 dye (emission wavelength of 690 nm). The core-shell nanoparticles (particle size ≈ 100 nm) retained the membrane proteins F4/80 and CCR2 (Western blot verification) and exhibited excellent biocompatibility (the CCK-8 assay showed 90% cell viability at high Fe concentrations). In vitro studies revealed threefold higher uptake of Fe3O4@M2 NPs in lipopolysaccharide-activated Raw 264.7 macrophages compared to uncoated particles (flow cytometry quantification), with 60% uptake inhibition in normal endothelial cells. In high-fat diet-induced *ApoE*^-/^^−^ mice, the probe was targeted and enriched in plaques via the enhanced permeability retention (EPR) effect and the CCR2-CCL2 axis, with NIR imaging showing 2.5-fold higher fluorescence intensity in the aortic arch than in controls (*p* < 0.001) and MRI demonstrating a 30% reduction in T₂ signal intensity within plaques (*p* < 0.05). Notably, the probe minimized immunogenicity (serum IgM/IgG levels decreased by 40%–60% after the second injection, *p* < 0.01) and did not induce liver/kidney dysfunction (ALT, AST, CRE levels comparable to controls) or organ pathology (confirmed by H&E staining). This work presents a novel nano-tool with high targeting specificity, low immunogenicity, and multimodal imaging capacity for non-invasive AS diagnosis, offering both real-time visualization of the inflammatory cascade and molecular-level dynamic analysis for evaluating anti-inflammatory therapy efficacy and plaque stability.

### 3.4. Targeting of fluorescent probes to other atherosclerotic process products

In addition to the aforementioned targets, significant advancements have been achieved in recent years in designing fluorescent probes for nontraditional biomarkers in the AS pathological process. The highly selective expression of E-selectin renders it an ideal target for drug delivery, minimizing its non-specificity to normal tissues and enhancing therapeutic precision. Li et al. developed E-selectin-targeting peptide (Esb peptide)-modified liposomes (T-AC-Lipo) via a thin film hydration-ultrasonic method and co-loaded them with atorvastatin calcium (Ato) and curcumin (Cur) for synergistic AS treatment (Li 2019). Experiments have demonstrated that the Esb peptide significantly enhances liposome uptake through specific binding to E-selectin, which is highly expressed on inflamed endothelial cells, resulting in lesion enrichment via receptor-mediated endocytosis. The dual-drug-loaded system synergistically inhibited the expression of adhesion molecules (E-selectin and ICAM-1 mRNA expression decreased by 59.38% and 34.42%, respectively) and inflammatory factors (IL-6, MCP-1). Meanwhile, curcumin effectively mitigated Ato-induced cytotoxicity, increasing cell viability from 55.72% to 70.12%. In *ApoE*^-/^^−^ mouse models, targeted therapy reduced the aortic plaque area from 61.58% in the control group to 17.14% and significantly reduced plasma total cholesterol and low-density lipoprotein levels by inhibiting monocyte migration and foam cell formation. This study revealed that the nano-targeted co-delivery system regulates the inflammatory microenvironment and lipid metabolism through multiple pathways, providing a highly efficient and safe new strategy for AS treatment.

Integrin αvβ3 is highly expressed on the surface of endothelial cells, macrophages, and neutrophils within atherosclerotic plaques but is expressed at low levels in normal tissues, making it an ideal target to circumvent non-specific damage to healthy cells. Shi et al. developed cRGD peptide-modified targeted liposomes (cRGD-SVT-Lipo) for efficient delivery and precise treatment of the anti-atherosclerotic drug Sivelestat (SVT) (Shi 2024). Prepared via the thin-film dispersion method using 1,2-dioleoyl-sn-glycero-3-phosphocholine (EPC) and cholesterol as the lipid bilayer skeleton, the liposomes were surface-functionalized with DSPE-PEG2000-cRGD, yielding particles with an approximate size of 100  nm, an 80% encapsulation efficiency, and in vitro release profiles demonstrating 24-hour sustained release (30% cumulative release). The probe design capitalized on the specific binding of the cRGD peptide to integrin αvβ3 on plaque endothelial cells and neutrophils, leveraging neutrophil chemotaxis for dual targeting: direct binding to the plaque vasculature and hitchhiking via migrating neutrophils to inflammatory sites. In vitro studies showed that cRGD modification increased neutrophil uptake efficiency by more than two-fold, significantly inhibiting phorbol 12-myristate 13-acetate (PMA)-induced neutrophil extracellular trap (NET) formation and elastase activity. In the *ApoE*^-/^^−^ AS mouse model, in vivo imaging and tissue section analysis confirmed that cRGD-SVT-Lipo accumulated in aortic plaques with a threefold higher intensity than unmodified liposomes. After three weeks of treatment, the plaque area decreased by 30%, the collagen content doubled, the number of elastic fibers decreased by 50%, and Cit-H3-labeled NET formation within plaques was suppressed. Pharmacokinetic analyses revealed a prolonged SVT half-life and increased area under the curve (AUC), while H&E staining of major organs demonstrated no toxicity. This study establishes a novel paradigm for neutrophil-targeted nanomedicine for treating inflammation-related diseases, integrating dual-targeting strategies with sustained drug release for enhanced therapeutic precision and safety.

Additionally, PO_4_^3^^−^ resulting from protein phosphorylation has emerged as a research focal point. Wen's team developed a bifunctional probe, I^3^^−^-RhB@PCN-224, based on a metal‒organic framework (MOF) (Wen et al. 2023). This probe enables dual-wavelength simultaneous detection (λex = 417/550 nm) through specific coordination of Zr^4+^ with PO_4_^3^^−^ and glucose recognition by the I^3^^−^ RhB moiety. The detection limits of PO_4_^3^^−^ and glucose were as low as 0.28 and 0.15 mM, respectively. In an early-stage AS mouse model, two-photon imaging revealed significantly increased PO_4_^3^^−^ and glucose levels in the thoracic aorta and organs, with a fluorescence intensity 2.3-fold higher than that in normal controls, achieving imaging depth down to the tissue’s micron-scale architecture.

Recently, Zhang et al. identified a novel mechanistic target, whereby ceramide species, including C16:0 and C18:0, exacerbate AS by directly binding to the G protein-coupled receptors (GPCRs) CYSLTR2 and P2RY6 on the cell membrane, thereby activating the Gq signaling pathway and the NLRP3 inflammasome (Zhang 2025). Cryo-electron microscopy showed that ceramide specifically recognizes the fatty acid chain of CYSLTR2 through its hydrophobic pocket, triggering receptor conformational changes and activating downstream inflammatory responses. Functional studies demonstrated that genetic knockout or pharmacological inhibition of these two receptors significantly reduced atherosclerotic plaque formation and suppressed inflammatory cell infiltration in mice fed a high-cholesterol diet; in chronic kidney disease models, receptor antagonists alleviated ceramide-induced plaque deterioration without affecting cholesterol levels. Clinical data have shown that plasma ceramide levels are significantly elevated in chronic kidney disease patients with coronary heart disease and are positively correlated with the severity of coronary artery stenosis. This study elucidates a previously unrecognized mechanism of ceramide-mediated inflammation via GPCR receptors, providing a theoretical foundation for the development of anti-atherosclerotic drugs that target CYSLTR2/P2RY6, particularly for chronic kidney disease patients, whose efficacy is limited compared with that of traditional therapies.

## 4. Application of fluorescent probes in the diagnosis and treatment of atherosclerosis

Currently, the application of fluorescent probes in the diagnosis and treatment of AS has focused primarily on three key aspects. The first involves early plaque detection: early identification of atherosclerotic plaques and accurate assessment of their developmental stage are of paramount importance, as timely intervention may effectively preclude disease progression. The second pertains to precise intraoperative localization: surgical intervention represents a critical modality, and precise localization of lesion sites alongside assessment of plaque stability can provide substantial guidance for surgical decision-making. The third aspect is therapeutic efficacy monitoring: via dynamic monitoring of anti-AS drug efficacy, clinicians can optimize treatment strategies and enhance therapeutic outcomes. Integrating these three dimensions may effectively address the progression of AS and facilitate patient management. Currently available fluorescent probes that have reached the clinical stage are summarized in Table 2. Among these, indocyanine green (ICG) and methylene blue have gained widespread clinical acceptance, while 5-aminolevulinic acid (5-ALA) and OTL38 have received regulatory approval. In contrast, Bevacizumab-800CW, BLZ−100 (tozuleristide), and LUM015 remain under clinical investigation. Compared with these clinically advanced agents, many studies are still in the exploratory phase. Representative examples of in vivo imaging studies performed in murine models are provided in Table 3.

**Table 2.** Fluorescent probes entering the stage of human experiment.

Probe name	Type	Main targets/mechanisms	Clinical application fields	Stage of development
Indocyanine green (ICG)	Nontarget	EPR effect, hepatobiliary metabolism	Hepatobiliary, cardiovascular, tumor, lymph, plastic surgery	Approved clinical application
Methylene blue	Nontarget/Weak targeting	Parathyroid specific uptake	Parathyroid Glands, sentinel lymph nodes, urinary system	Approved clinical application
5-Aminolevulinic acid (5-ALA)	Metabolic activation	Heme synthesis pathway	Glioma, bladder cancer	Approved clinical application (Europe and America)
OTL38	Target	Folate receptor-α(fra)	Ovarian cancer, lung cancer	FDA approval (Ovarian cancer)
Bevacizumab-800CW	Target (antibody)	VEGF	A variety of solid tumors (head and neck cancer, breast cancer, etc.)	Clinical trial
BLZ−100 (Tozuleristide)	Target (polypeptide)	Tumor extracellular matrix	Brain Tumors (Adults and Children)	Clinical trial
LUM015	activation of enzymes	Cathepsin	Breast cancer, soft tissue sarcoma	Clinical trial

**Table 3.** In vivo imaging data of mice in some articles.

Author	Imaging system	Excitation filter	Probe metering	Size of animal
Kai Wang et al. (Wang 2022)	Maestro EX fluorescence imaging system	–	100 uL, 4.5 mg kg^−1^	35
Xinyu Xu et al. (Xu 2025)	Home-made small animal imaging system/IVIS Lumina XR	710 nm, 808 nm, 940 nm	100 uL, 150 μM	–
Hui Wang et al. (Wang et al. 2023)	IVIS Lumina III In Vivo Imaging System	420 nm	100 uL, 100 μM/25 g	24
Zhuo Ye et al. (Ye 2022)	Azure Imaging System	535 nm	100 uL, 30 μg/25 g	12
Mangmang San et al.(Sang 2021)	living imaging system	488 nm	100 uL, 10 μM	–
Jinrong Zheng et al. (Zheng et al. 2021)	IVIS Spectrum system (PerkinElmer, Austria)	488 nm	100 uL, 10 μM	12
Feng Ren et al. (Ren 2020)	NIR II Imaging System (Serious II 900–1700)	LP1400 nm	200 uL, 20 mg/kg	–

### 4.1. Early detection of atherosclerotic plaque

**Figure 6.**
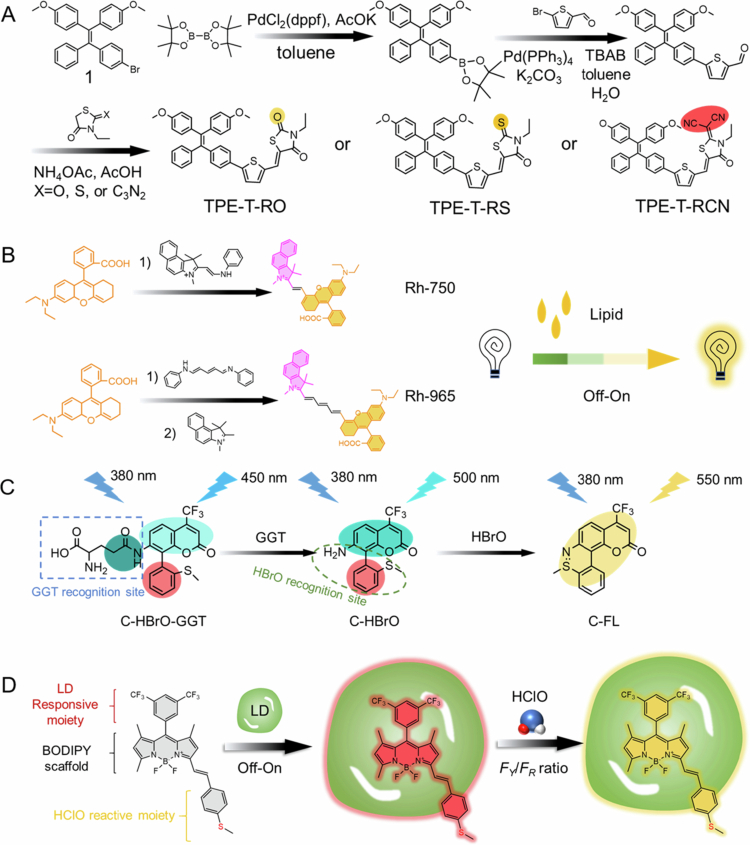
(a) The synthetic routes and fluorescent group diagrams of TPE-T-RO, TPE-T-RS, and TPET-RCN (Wang 2022). (b) The synthesis route, chemical structure of Rh-750 and Rh-965, and the schematic diagram of their fluorescence response effect (Xu 2025). (c) A schematic diagram of the chemical structure of C-HBrO-GGT and its response mechanism to GGT and HBrO (Wang et al. 2023). (d) A schematic diagram of iSHERLOCK for ‘off-on’ and ratiometric detection of LDs and HClO. FY and FR represent the fluorescence intensity (FI) in the yellow and red channels, respectively (Ye 2022).

Currently, the dominant methods for early atherosclerotic plaque detection depend primarily on specific targeting sites within lesion areas. Specifically, the first approach involves the design of AIE-active nanoprobes with lipid-targeting specificity. Wang et al. developed a high-brightness AIE nanoprobe through a molecular engineering strategy (Figure 6a) (Wang 2022). By optimizing the electron donor-acceptor structure of rhodanine derivatives, the study significantly enhanced the photophysical properties of the probe. The results demonstrate that the AIE luminophore (TPE-T-RCN), featuring dicyanomethylene-modified rhodamine as the electron-withdrawing unit, has an exceptional molar extinction coefficient (*ε* = 1.2 × 10^5^ M^−1^cm^−1^), a photoluminescence quantum yield (ΦPL = 42%), and red-shift spectral characteristics (λabs/λem = 520/650 nm). The probe enabled highly sensitive detection of atherosclerotic plaques in an in vitro model (detection limit: 0.5 nM) and was successfully applied to high-throughput screening of anti-atherosclerotic drugs. Notably, the probe exhibited a 3.2-fold higher signal-to-noise ratio than the traditional probe.

The second direction involves the development of lipid-responsive NIR-II fluorescent probes. Xu's team designed an NIR-II (1000−1700 nm) fluorescent probe, Rh-965 (Xu 2025), by introducing a *π*-conjugated extended polyene bridge structure into the hemicyanine dye Rh-750, which shifted the emission wavelength from NIR-I (750 nm) to NIR-II (965 nm) (Figure 6b). Via intramolecular motion restriction, the probe exhibited a 25-fold increase in fluorescence intensity within a lipid-enriched microenvironment, achieving a detection limit of 4.16 μg/mL for liposomes. In vivo experiments demonstrated that carotid plaques in *ApoE*^-/^^−^ mice could be localized within 3 m of local administration. Imaging revealed that the fluorescence intensity of the plaque area was 1.44-fold higher than that of normal tissue (*P* < 0.01), with an ex vivo tissue signal-to-background ratio of 7.84 and a penetration depth of 8 mm. Histopathological analysis confirmed its biosafety with an LD_50_ > 200 mg/kg), providing a new scheme for precise intraoperative navigation.

For precise localization, Wang et al. constructed a dual-responsive probe, C-HBrO-GGT, that targets gamma-glutamyl transpeptidase (GGT) and hypobromous acid (HBrO), achieving ultra-early diagnosis of AS through a sequential activation mechanism (Figure 6c) (Wang et al. 2023). The probe undergoes sequential enzymatic and chemical activation: first, GGT-mediated hydrolysis exposes a primary amino group (kcat/Km = 1.8 × 10^3^ M^−1^ s^−1^), followed by a specific reaction with HBrO to generate the red fluorescent product C-FL (λem = 620 nm). In vitro studies showed that GGT and HBrO induced 18-fold and 29.78-fold increases, respectively (*R*^2^ = 0.989), with detection limits of 0.076 U/L for GGT and 0.32  μM for HBrO. Mechanistic analyses revealed that ox-LDL stimulation increased GGT activity by 2.70-fold and HBrO levels by 2.97-fold in foam cells, involving the CLC-1 chloride channel-mediated Br^-^ influx and catalase oxidative inactivation pathways. In the *ApoE*^-/^^−^ mouse model, the probe detected abnormal GGT (4.56-fold) and HBrO (2.64-fold) signals 4−8 prior to plaque formation, preceding the expression changes of traditional biomarkers such as CD40.

To increase targeting sensitivity, Ye's group synthesized the dual-mode probe iSHERLOCK (MTB-B-CF3) with lipid droplet (LD) targeting and hypochlorous acid (HClO) responsiveness via acid-catalyzed condensation and the Knoevenagel reaction using the BODIPY 493/503 fluorescent skeleton (Figure 6d) (Ye 2022). The probe exhibited a fluorescence quantum yield of 0.68 in a low-polarity environment, achieving a detection limit of 157  ng/mL for LDs with a response time < 60 s. It displays high selectivity for HClO (Kd = 87 nM), with a detection sensitivity 5.3-fold higher than that of traditional probes. In the AS model, the probe enables dynamic monitoring of lipid deposition and oxidative stress through ‘off-on’ and ratio-signal dual modes. In vitro plaque imaging revealed a target-area signal-to-noise ratio of 12.7:1. Although its tissue penetration depth (~3 mm) and water solubility (logP = 2.9) require further optimization, its modular design offers a viable pathway for clinical transformation.

### 4.2. Guiding surgical treatment

The role of intraoperative fluorescence imaging for precise guidance in AS surgical treatment has gained increasing prominence. The central objective of intraoperative fluorescence imaging is to achieve real-time visualization of plaque boundaries through the coordination of targeted probes and highly sensitive optical systems. Marcella's team developed a near-infrared fluorescence (NIRF) catheter system employing protease-activated probes (e.g. MMP-9-responsive probes), leveraging the low tissue absorption and low autofluorescence characteristics within the 650−950 nm spectral window (Marcella et al. 2010). The intraoperative plaque-to-background signal ratio (TBR) increased to 6.8 ± 1.9 (compared to only 1.3 ± 0.3 in the control group). In contrast, the commonly used VCAM-1-targeted ultrasound contrast agent reached a TBR of only 4.7 ± 1.2 in the rabbit atherosclerosis model. By integrating with a clinical optical tomography platform (750 nm excitation/780 nm collection), the system achieves real-time discrimination of plaque/normal vascular segments in rabbit models. The TBR is highly correlated in vivo and ex vivo (*r* = 0.82), with negligible blood interference (signal attenuation < 15%).

**Figure 7.**
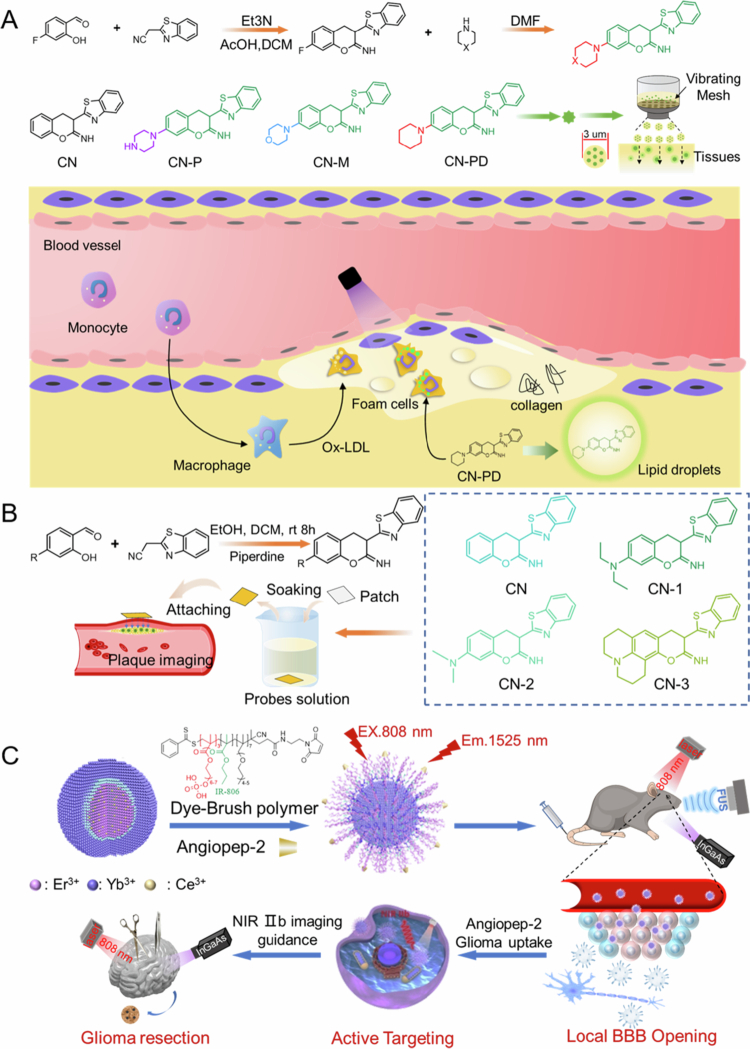
(a) The synthesis route of CN-P, CN-M, and CN-PD probes, the form of probe infiltration, the evolution of foam cells in the plaque and the corresponding probe recognition mechanism, and the fluorescence imaging process of using in situ foam cell-specific probes to soak the plaque during surgery (Sang 2021). (b) The synthesis route of CN-1, CN-2, and CN-3 probes and the fluorescence imaging process of using in situ foam cell-specific probes to soak plaques during surgery (Zheng et al. 2021). (c) Synthesis of Er-DCNPs-Dye-BP-ANG probe and its multi-mode collaboration. NIR IIb fluorescence imaging guides the resection of deep and small-sized in situ gliomas (Ren 2020).

Sang et al. developed the coumarin derivative probe CN-PD, which achieves a lipid-specific fluorescence response via piperidinyl modification (>10-fold fluorescence enhancement) (Figure 7a) (Sang 2021). Its 3 μm atomized particles enable in situ plaque labeling within 5 min, combined with visible light fluorescence imaging (λem = 520 nm) to accurately delineate plaque boundaries (e.g. 50 μm edge resolution), reducing intraoperative systemic probe exposure by 70%. The further developed CNN2-B probe simultaneously monitors lipid accumulation (LOD = 72.6 ng/mL) and oxidative stress (LOD = 1.3 nM) through the ONOO-triggered borate deprotection mechanism, demonstrating dual signal amplification in the plaque area (exhibiting 8.2-fold higher fluorescence intensity than normal tissue) (Figure 7).

Subsequently, they designed the coumarin-based lipid-activated fluorescent probe CN-N2, optimizing its optical properties via diethylamine group incorporation, whereby fluorescence was quenched in aqueous environments but significantly enhanced (6.6-fold) in lipid environments due to restricted molecular rotation (Figure 7b) (Zheng et al. 2021). The probe emitted visible light at 520 nm with a fluorescence quantum yield of 0.35, achieving a liposome detection limit as low as 200  μg/mL and a response time <1 m. CN–N2 highly colocalized with lipid droplets in A549 cells (PCC = 0.94) and penetrated 1.5 mm of adipose tissue, with the fluorescence intensity reaching a 36-fold background within 5 min. In an AS mouse model, the probe visualized carotid plaques with 90% luminal diameter stenosis at a 50 μM concentration. This study provides a rapid, highly sensitive visualization tool for precise intraoperative localization of atherosclerotic plaques, with potential for clinical translation.

Ren et al.'s breakthrough in NIR-II imaging (λem = 1525 nm) provides novel insights for AS surgery: through an erbium-based rare earth nanoparticle-based energy cascade downconversion strategy combined with focused ultrasound-mediated blood–brain barrier opening technology, multimodal localization of deep-seated plaques is achieved (axial resolution of MRI-NIR-II fusion imaging <2 mm) (Figure 7c) (Ren 2020). Although intraoperative fluorescence technology still faces challenges such as the regulation of probe metabolic kinetics (e.g. CN-PD half-life of only 2.3 h) and multi-spectral signal crosstalk, its integration with robot-assisted surgical systems (e.g. NIR-II-guided robotic arm plaque resection accuracy of 0.1 mm) is propelling AS treatment toward a precise and minimally invasive paradigm.

### 4.3. Monitoring of drug efficacy

AS is the primary pathophysiological foundation of CVDs, exhibiting chronic inflammatory characteristics intimately related to plaque vulnerability. While existing drugs such as statins hinder AS progression by regulating lipid metabolism, a substantial gap persists in efficient drug screening techniques for dynamic changes in the plaque microenvironment, alongside arduous challenges in drug co-loading (Liu 2018). In recent years, fluorescent probe technology has emerged as an innovative tool for assessing the efficacy and dissecting the mechanism of anti-AS drugs, owing to its high sensitivity, specific targeting capability, and real-time visualization attributes.

For example, Jiang's team developed a Neu-NP probe via a biomimetic strategy that conjugates neutrophil proteins with indocyanine green (ICG)-loaded nanoparticles to leverage the natural homing ability of neutrophils to inflammatory sites for precise targeting of high-risk plaques (Jiang et al. 2022). In the *ApoE*^-/^^−^ mouse model, Neu-NPs enabled dynamic monitoring of plaque inflammation regression after rosuvastatin treatment via NIR-II imaging: the fluorescence signal intensity in the drug-treated group was significantly lower than that in the control group (a 60% reduction), intuitively reflecting the drug’s inhibitory effect on macrophage infiltration and pro-inflammatory factor expression. This in vivo imaging technology based on targeted probes not only overcomes the static limitations of traditional histological analysis but also achieves longitudinal evaluation of drug efficacy by quantifying signal differences.

Li et al. developed the GSH-responsive fluorescent probe CBF, incorporating coumarin as the fluorophore and chloropropionate as the reactive moiety (Li 2020). Three analogs (CBF1–CBF3) were designed through structural optimization by altering the halogen substituents and alkyl chain lengths. The CBF3 probe exhibited exceptional performance, with a detection limit for GSH as low as 9.2 nM, a linear response of 0−50 μM (*R*^2^ = 0.9967), and a fluorescence quantum yield of 0.85. At equimolar concentrations, the selectivity of CBF3 for GSH was -fold greater than that of Cys and 13-fold greater than that of Cys and Hcy, respectively, and CBF3 outperformed CBF1 and CBF2. In HepG2 cells, CBF3 clearly visualized the GSH distribution, with fluorescence markedly attenuated after treatment with *N*-ethylmaleimide (GSH inhibitor), confirming its specificity. In Caenorhabditis elegans, GSH detection via CBF3 enables drug efficacy evaluation at the in vivo level, minimizing discrepancies between in vitro assays and in vivo outcomes while providing technical support for in vivo drug targeting and safety assessment. This study demonstrated that fluorescent probes facilitate accurate detection, mechanism analysis, and in vivo evaluation in drug screening, advancing their utility through methodological innovation and offering efficient tools for discovering metabolic pathway-targeted therapeutics.

Currently, fluorescent probe technology has demonstrated three core advantages in AS drug research and development: (1) Spatiotemporal dynamic monitoring: real-time tracking of dynamic changes in the plaque microenvironment (e.g. ROS levels and MMP-9 activity) following drug treatment via in vivo imaging (Liu et al. 2018). (2) Targeted precision: specific recognition of pathological markers (e.g. CD47, IL-1β) achieved through antibody or biomimetic modification. (3) Theranostic integration: certain probes (e.g. drug-loaded liposome–DiR complexes) enable simultaneous drug delivery and efficacy feedback. With the development of dual-responsive probes (e.g. pH/enzyme activity co-responsive systems) and artificial intelligence-assisted signal analysis technologies, this field is rapidly advancing toward personalized precision medicine, offering a new paradigm for overcoming bottlenecks in AS treatment.

For clinical applications, an SNR greater than 10 is generally needed during the optimal imaging window to support decision-making in specific clinical tasks. The pharmacokinetic profile should be characterized by a moderate half-life and rapid blood clearance, typically within 1–4 h post-injection. Furthermore, the agent must demonstrate a high safety standard – exhibiting no acute toxicity, no phototoxicity, minimal risk of long-term retention, and a well-defined excretion pathway. The incidence of adverse reactions at recommended clinical doses should be very low. Therefore, significant challenges remain in advancing fluorescent probe research to the clinical stage.

## 5. Conclusion and foresight

Fluorescent probe technology has emerged as an important tool in the pathological mechanism analysis, diagnosis, and treatment evaluation of AS owing to its high sensitivity, dynamic imaging, and multi-parameter analysis capabilities. Through targeted design, such as reactive oxygen species-responsive and enzyme-activated probes, researchers can detect key biomarkers (e.g. reactive oxygen species (ROS) and matrix metalloproteinases (MMP)) in atherosclerotic plaques with nanomolar sensitivity. When combined with NIR-II imaging, these probes enable dynamic, high-resolution visualization of plaques with centimeter-level penetration depth in vivo, thereby elucidating the spatiotemporal dynamics of pathological events such as macrophage polarization and lipid core expansion. In addition, the development of ratiometric probes and ‘double-lock’ logic-gated probes significantly reduces the interference of complex microenvironments (pH gradients and multienzyme coexistence). These systems employ dual-channel signal correction or dual-activation mechanisms to improve the detection specificity and achieve levels exceeding 95%. However, current technologies still face challenges such as insufficient biocompatibility (the probe is highly toxic and difficult to metabolize (Abiodun Daramola 2024; Li et al. 2024), clinical transformation barriers (human autofluorescence interference, vascular anatomical differences (Chen et al. 2023). For example, certain probes have failed in clinical trials because of a 70% drop in the signal-to-noise ratio or because the positioning deviation exceeds 40% due to off-target activation. To overcome these challenges, future research should focus on multidisciplinary strategies. (1) Multimodal probes such as fluorescence-MRI-PET composite probes should be developed to combine the advantages of preoperative positioning and intraoperative navigation. (2) Construction of intelligent response systems, such as DNA origami-based or nanologic-gate architectures, to enable collaborative recognition of multiple biomarkers, thereby improving the diagnostic specificity of vulnerable plaques (the experimental model has reached 92%). (3) Optimization of nanodelivery systems using biomimetic carriers or ultrasound-triggered release mechanisms to increase targeted probe accumulation (e.g. the enrichment of membrane-coated nanoparticles results in an 8-fold increase in plaque enrichment). (4) Integration of artificial intelligence algorithms such as 3D U-Net to realize automatic quantitative analysis of fluorescence images and control the error rate of lipid core recognition to be less than 5%. Although fluorescent probes have shown potential in molecular typing diagnosis and precise intervention, successful clinical transformation will require systematic resolution of key problems, including pharmacokinetic optimization, establishment of a standardized evaluation system (including signal quality, accurate targeting, efficient response, safe and available, stable and reliable) and large-scale production. Moreover, it is necessary to strengthen and improve the regulatory system to promote the transition of cardiovascular diagnostics and therapeutics into the era of molecular precision.

## Data Availability

Data sharing is not applicable to this article, as no new data were created or analyzed in this study.
